# Ammonia Generation System for Poultry Health Research Using Arduino †

**DOI:** 10.3390/s21196664

**Published:** 2021-10-07

**Authors:** Dan Hofstetter, Eileen Fabian, A. Gino Lorenzoni

**Affiliations:** 1 Department of Agricultural and Biological Engineering, The Pennsylvania State University, University Park, State College, PA 16802, USA; efw2@psu.edu; 2 Department of Animal Science, The Pennsylvania State University, University Park, State College, PA 16802, USA; agl20@psu.edu

**Keywords:** ammonia, Arduino, controls, gas sensor, MQ-137, NH_3_, poultry, ultrasonic humidifier

## Abstract

An ammonia gas (NH_3_) generator was developed to maintain a set concentration of ammonia gas in a controlled environment chamber to study poultry physiological responses to sustained elevated levels of ammonia gas. The goal was to maintain 50 parts per million (ppm) of ammonia gas in a 3.7 m × 4.3 m × 2.4 m (12 ft × 14 ft × 8 ft) controlled environment chamber. The chamber had a 1.5 m^3^/s (3000 cfm) recirculation system that regulated indoor temperature and humidity levels and a 0.06 m^3^/s (130 cfm) exhaust fan that exchanged indoor air for fresh outdoor air. The ammonia generator was fabricated by coupling ultrasonic humidifiers with an Arduino-based microcontroller and a metallic oxide MQ-137 ammonia gas sensor. Preliminary evaluation under steady conditions showed the average MQ-137 gas sensor accuracy was within 1.4% of the 65.4 ppm concentration measured using a highly accurate infrared gas analyzer. Further evaluation was performed for a setpoint concentration of 50 ppm where ammonia generator reservoirs were filled with 2% or 10% ammonia liquid. For the system tested, it was found that two generators operating at the same time filled with 3.8 L (1.0 gallon) of 2% ammonia cleaning liquid each (7.6 L or 2.0 gallons total) could maintain a gas level of 49.45 ± 0.79 ppm in the chamber air for a duration of 30 h before refilling was required. One generator filled with 3.8 L of 10% ammonia cleaning liquid could maintain 51.24 ± 1.53 ppm for 195 h. Two ammonia generators were deployed for a six-week animal health experiment in two separate controlled environment chambers. The two ammonia generators maintained an average ammonia concentration of 46.42 ± 3.81 ppm and 45.63 ± 4.95 ppm for the duration of the experiment.

## 1. Introduction

Ammonia (NH_3_) is a natural component of the bacterial fermentation occurring in fecal matter and urine [1]. Animal confinement and low levels of ventilation cause ammonia gas to accumulate in animal barns [2,3]. Ammonia levels as low as 50 to 75 ppm can reduce the productivity levels of modern commercial broiler chickens [4]. High levels of ammonia can produce inflammation in the upper respiratory tract of poultry, making the respiratory system susceptible to viral and bacterial infections [5].

Ammonia is rated Immediately Dangerous to Life or Health (IDLH) for human workers at a concentration of 300 ppm, and has a permissible exposure limit (PEL) of 50 ppm as a time weighted average (TWA) by the Occupational Safety and Health Administration [6]. The National Institute for Occupational Safety and Health [7] has a recommended exposure limit (REL) of 25 ppm TWA and 35 ppm short-term exposure limit (STEL). Animal welfare guidelines for the indoor ammonia concentration of egg-laying flocks and broiler hens are typically below 10 ppm [8,9] at bird level, with recommended limits of 20 ppm [10,11] and short-term maximums not exceeding 25 ppm [8,9,12] during extreme weather events.

The concentration of ammonia inside a poultry house is highly variable. Housing types, such as aviary, caged, high rise, and free-range, affect bird stocking density and manure handling options. Manure accumulates for different durations depending on removal intervals [13], and indoor ammonia concentrations are higher immediately before the removal of manure from the barn [14]. The ammonia production rate in free-range housing also depends on litter pH and moisture content [15,16,17]. Ventilation type and air exchange rate greatly affect indoor ammonia concentration [16,18] and temperature. Ammonia volatilization rates in poultry barns are higher during periods of warm weather than cold [15,19], and diurnal variations have also been reported [3,20]. Ammonia concentrations are higher in naturally ventilated barns [21], and minimum winter ventilation rates in mechanically ventilated barns typically result in higher indoor ammonia concentrations during cold weather [14,16,19]. Since it is not possible to tightly control the ambient conditions in poultry barns, animal health studies that expose poultry to a defined concentration of ammonia gas are usually performed inside controlled environment chambers.

Previous work has been done to quantify the effect of different levels of ammonia gas on poultry health. Most efforts have been directed towards administering the ammonia using compressed gas cylinders with dosing controlled by a flow meter [4,22,23,24,25,26,27,28,29]. Work with species other than chickens (pigs, rabbits, rats, and guinea pigs) has also been conducted using a similar technology [22,25]. There are three challenges that affect most of the literature dealing with the effect of ammonia on animal health. The first challenge is the lack of technology to frequently monitor the concentration of ammonia. Few studies monitor the ammonia concentration continuously [22,26,29], with most studies measuring the ammonia concentration one to three times daily [4,23,24,27,28] or only at the beginning and end of an experiment [25], while others do not report this information [30]. For this reason, information regarding the variability of the concentration of ammonia attained during an experiment is not uniformly reported in the current literature.

The second challenge is in relation to the lack of technology to compensate for variations in the concentration of ammonia that may occur during the course of an experiment. Since most studies supply ammonia gas at a fixed delivery rate using flow meters, the ammonia concentration could change if conditions inside the chamber change. A reduction in ventilation air flow rate due to clogged inlet air filters would cause the indoor ammonia concentration to increase above the desired level. Opening the doors of the chambers or animal rooms to attend the animals and service the equipment would cause the concentration of ammonia to decrease. Failing to immediately compensate for variations in the concentration of ammonia will unavoidably result in a lack of control of the applied treatment.

The third challenge of previously used ammonia supply systems is safety. Compressed anhydrous ammonia is toxic if inhaled and corrosive upon contact, potentially causing severe skin burns, frostbite to skin, and eye damage [31,32]. Most studies do not report safety devices to deal with accidental leakage of the gas. Electromechanical cutout valves that stop the flow of gas in case of a power failure have been used [19,20]. However, even with a cutout valve, there would still be risk to human personnel when making connections to the gas cylinder, in case of leakage from cracked hoses, or from partial power loss only affecting the ventilation system.

Additionally, the equipment required for ammonia supply systems using compressed gas cylinders such as hoses, pressure regulators, and flow meters and the volume of ammonia gas needed for long durations in multiple chambers has a high cost. The cost associated with the gas cylinders and the risk to human personnel and birds derived from accidental leaks from the compressed gas system prompted a search for a safer and more economical alternative. Previous work by the authors demonstrated the feasibility of generating ammonia gas from ammonia liquid using ultrasonic humidifiers controlled by an Arduino-based microcontroller in response to real-time gas sensor measurements [33,34]. The goal of this work was to develop a safe, affordable, and reliable method to generate and maintain a defined concentration of ammonia gas during animal research studies.

### Instrument Technology

The selection of an ammonia gas sensor for real-time feedback to control an ammonia generator was based on the following design requirements: to make real-time measurements, the sensor should have a fast response time (on the order of seconds); the sensor should be capable of continuous operation in the presence of levels of ammonia gas between 0 and 100 ppm for extended durations of time; the sensor should not be adversely affected by high levels of dust and humidity; the sensor should be small, with low power requirements, and inexpensive.

Many types of ammonia gas sensing technologies have been described comprehensively by Kwak, et al. [35], and in-depth comparisons have been made between solid-state and optical ammonia sensors [36]. Optical ammonia gas sensors typically work by measuring the light absorbed by the gas as it passes through an optical chamber. Optical sensors are capable of very accurate measurements, but the optical flow chamber often contains lenses or mirrors that can be fouled or damaged in very dusty and humid environments, making them unsuitable for continuous use in farm environments.

Commercially available ammonia monitors suitable for use in poultry housing typically have a high cost; however, low-cost sensors have become available in recent years. Electrochemical gas sensors are commonly used to detect ammonia in handheld gas monitors costing roughly $400 to $3500. These sensors contain electrodes that are surrounded by a liquid electrolyte. When the ammonia gas comes into contact with the sensing electrode, an electrochemical reaction occurs that utilizes part of the available liquid electrolyte, shortening its lifespan [37]. For this reason, the sensors used in handheld gas monitors are not well suited for the continuous monitoring of ammonia gas in poultry houses. The liquid electrolyte may also dry out if the relative humidity level of the air is very low [37], which is common during winter months when supplemental heat is added to poultry houses. Replacement sensors are available but may cost as much as $500 each. A longer-life electrochemical sensor designed to be permanently mounted in animal barns has recently been produced by Dräger (DOL 53, Drägerwerk AG & Co. KGaA, Lübeck, Germany), with an advertised working life span of three years, but the cost of one of these sensors was around $1000 at the time of this study.

Metallic oxide gas sensors were first developed in the 1950s [38]. Over the years, the technology has advanced enough that small sensors can be manufactured for a low cost. These sensors contain a heated sensing element such as stannic oxide (SnO_2_) that changes resistance when exposed to different concentrations of target gases. Metallic oxide sensors are compact, react to gas concentration changes very quickly [39], and do not consume sensor material when exposed to target gases [37], making them suitable for the continuous monitoring of ammonia gas in animal facilities; However, metallic oxide gas sensors usually must be operated for an extended period of time, from several minutes to many hours, to heat the sensing element before stable measurements can be made. Metallic oxide gas sensors typically draw significantly higher electrical current than electrochemical sensors, which makes them unsuitable for use in battery-operated handheld instruments.

Both electrolytic and metallic oxide gas sensors are sensitive to changes in air temperature and humidity, which may alter sensitivity and change output readings [37]. Most commercial gas monitors have circuitry to adjust for changes in atmospheric conditions. Bare off-the-shelf sensors lack this extra circuitry, so using them requires additional signal processing to correct for changes in temperature and humidity, or the sensors should be used in tightly controlled environments.

## 2. Materials and Methods

### 2.1. Overview

A sensor array was developed from off-the-shelf components to work together in not only documenting and datalogging conditions but also to precisely control an ammonia generator for producing a set ammonia level in a controlled indoor environment. Suitable ammonia gas sensors were evaluated for accuracy in a controlled environment chamber. Ammonia gas generators were developed using ultrasonic humidifiers filled with store-bought ammonia cleaning liquid. Preliminary tests [33] were performed in a controlled environment chamber to determine the maximum concentration of ammonia gas produced from 2% (*v*/*v*) liquid ammonia using one or two ammonia generators operating at full speed continuously. Subsequent evaluations of the ammonia generation system [34] were conducted using 2% and 10% (*v*/*v*) strength liquid cleaning ammonia with one or two ultrasonic ammonia generators. After evaluation, the ammonia generators were deployed in two separate controlled environment chambers to continuously maintain a target ammonia gas concentration of 50 ppm for a six-week poultry health study.

### 2.2. Controlled Environment Chamber

Experiments were performed in a controlled environment chamber at the Poultry Education and Research Center (PERC) at Pennsylvania State University. The chamber had dimensions of 3.7 m × 4.3 m × 2.4 m (12 ft × 14 ft × 8 ft), and the walls, floor, and ceiling were stainless steel. Exhausted air was replaced by fresh outdoor air drawn into the room inlets by a continuously operating 0.06 m^3^/s (130 cfm) exhaust fan located in the ceiling in the back-left corner of the chamber. The air in the chamber was well-mixed as a result of a 1.5 m^3^/s (3000 cfm) air handling unit that recirculated air in the room to maintain temperature and humidity settings. Temperature could be controlled between 4 and 40 °C (±0.5 °C), and humidity was added to the chamber by a Nortec EL-50 steam humidifier (Nortec Humidity Ltd., Ottawa, ON, Canada). During the experimental timeline, chamber dehumidifiers were not functional, therefore excess humidity was not removed from the environment.

### 2.3. Air Quality Monitor and Datalogger

**Figure 1:**
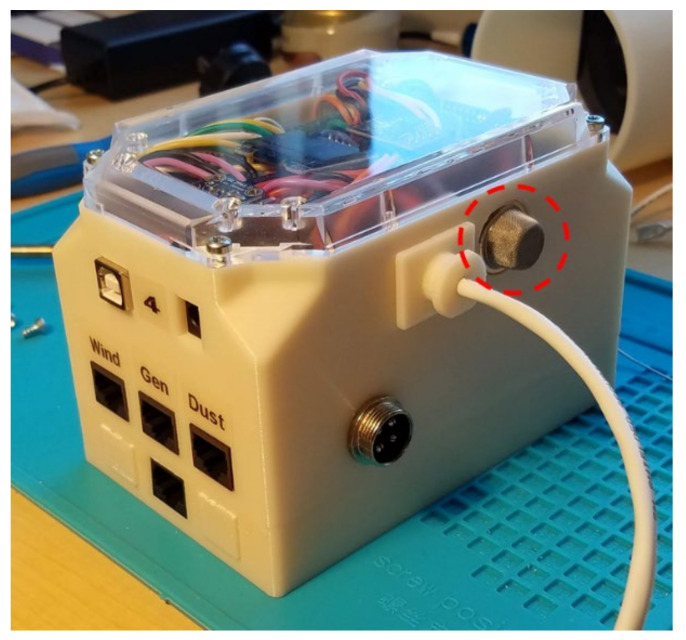
Custom environment monitor and datalogger (ammonia sensor shown in dashed circle).

A custom-built indoor air quality monitor and datalogger recorded data and controlled the concentrations of airborne ammonia gas in the chamber (Figure 1). An Arduino MEGA 2560 microcontroller (Elegoo, Inc., Shenzhen, China) recorded temperature, relative humidity, luminosity, carbon dioxide concentration, ammonia gas concentration, dust concentration, exhaust air velocity, and ammonia generator speed. A real-time clock module with backup battery provided the time and date. All measured data were logged to a 16 GB microSD card using a comma-separated value (.CSV) file format. A 2.4 cm (0.96 in.) LCD display showed measured temperature, humidity, ammonia, carbon dioxide, and dust concentrations. A Wi-Fi module was installed to allow data to be sent to a website for the real-time online monitoring of the controlled indoor environment. This paper is focused on the components of the control system related to automatic ammonia generation: the microcontroller, the ammonia gas sensor, and the ammonia generator.

### 2.4. Ammonia Sensor

A metallic oxide semiconductor (MOX) ammonia sensor (MQ-137, Sainsmart, Lenexa, KY, USA) was used to measure the ammonia gas concentration inside the controlled environment chamber. The ammonia sensor came equipped with a 10 kΩ load resistor (R_L_), but that resistor was removed from the sensor module and replaced because the sensor datasheet [40] calibration curves referred to a load resistor value of R_L_ = 47 kΩ.

### 2.5. Ammonia Generator

Ammonia gas was generated using modified ultrasonic humidifiers (Vicks model V4600, Kaz USA, Inc., Marlborough, MA, USA) filled with clear liquid ammonia. The cost for the ultrasonic humidifiers was approximately $40 per unit, and the cost for the ammonia cleaning liquid was $1.16 per 1.9 L (0.5 gallon) bottle for 2% *v/v* (Great Value brand, purchased at Walmart), or $5.99 for 3.8 L (1.0 gallon) for 10% *v/v* (Ace brand, purchased at Ace True Value Hardware).

**Figure 2:**
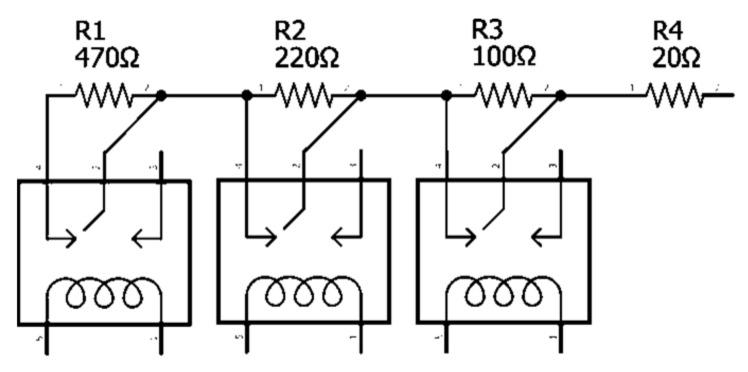
Humidifier speed control circuit schematic using relays to bypass resistors.

Each ultrasonic humidifier had a potentiometer that could be manually adjusted to change the humidifier output speed. The potentiometer varied the resistance from approximately 3 kΩ at the lowest speed to 20 Ω at the highest speed. During testing, it was determined that a resistance greater than 810 Ω resulted in the humidifier output being too low to be useful for ammonia generation. An automatic switching circuit was built using three 5 V relays in a 4-channel relay module that bypassed resistors in the circuit to create a variable resistor with discrete steps used to control the humidifier output speed (Figure 2). The resistors were soldered to a mini solder-able breadboard. The resistor values were chosen to create a nearly linear change in ammonia gas concentration with speed. This variable resistance was controlled automatically by the Arduino microcontroller. Table 1 shows the total resistance and relay states (Open or Closed) for each humidifier speed used.

**Figure 3:**
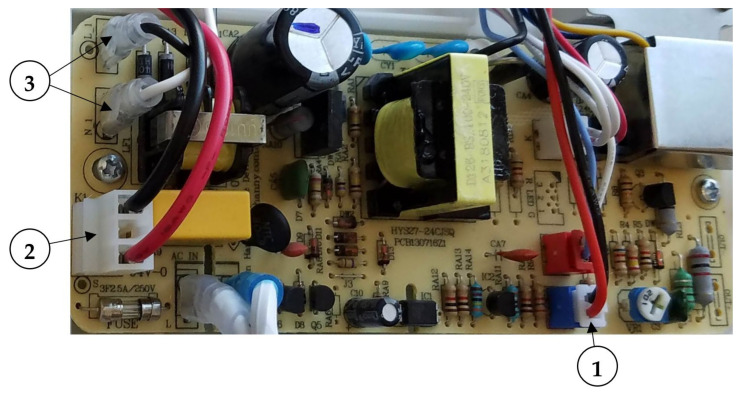
Plug locations for modification to main circuit board for the ultrasonic humidifier: 1. Speed control; 2. Power switch; 3. 120VAC connected to 5VDC power supply.

The stock humidifier speed control potentiometer was disconnected from the main circuit board (location 1 in Figure 3) and replaced with the output plug from the automatic switching circuit. The stock humidifier power switch, which was integral to the speed control potentiometer, was de-soldered and replaced by a fourth relay connected to the main circuit board (location 2 in Figure 3) to turn the ultrasonic humidifier on and off. The stock ultrasonic humidifier also contained a small resistive heater connected to blade terminals on the main circuit board that supplied 120VAC power (location 3 in Figure 3).

**Figure 4:**
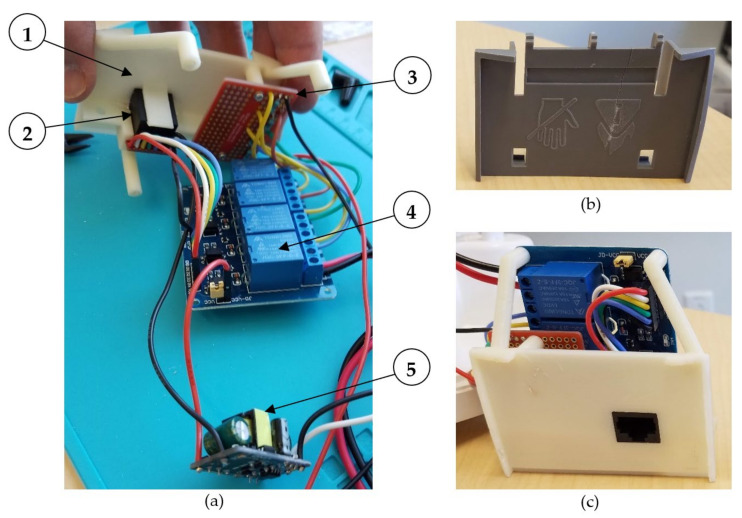
(**a**) Components used to automatically control the ultrasonic humidifier: (1) 3D printed mount; (2) RJ-45 jack; (3) resistor board; (4) 4-channel relay module (not fastened to the mount for illustration); and (5) 120VAC to 5VDC power supply module. (**b**) Resistive heater. (**c**) Assembled components.

The resistive heater was disconnected, removed from the humidifier, and replaced by (1) a custom 3D printed mount used to secure (2) an RJ-45 jack that connected the switching circuit to the microcontroller, (3) the solder-able breadboard with resistors, and (4) the 4-channel relay module (items 1 to 4 in Figure 4). The relays (Tongling P/N JQC-3FF-S-Z, 5 V coil, 10 A @ 250VAC) had a rated coil current of 71.4 mA, which was higher than the maximum supply current of 40 mA per input/output (I/O) pin for the Arduino MEGA 2560 microcontroller, so (5) an external 5VDC 700 mA power supply module was used to power the relay coils (item 5 in Figure 4).

**Figure 5:**
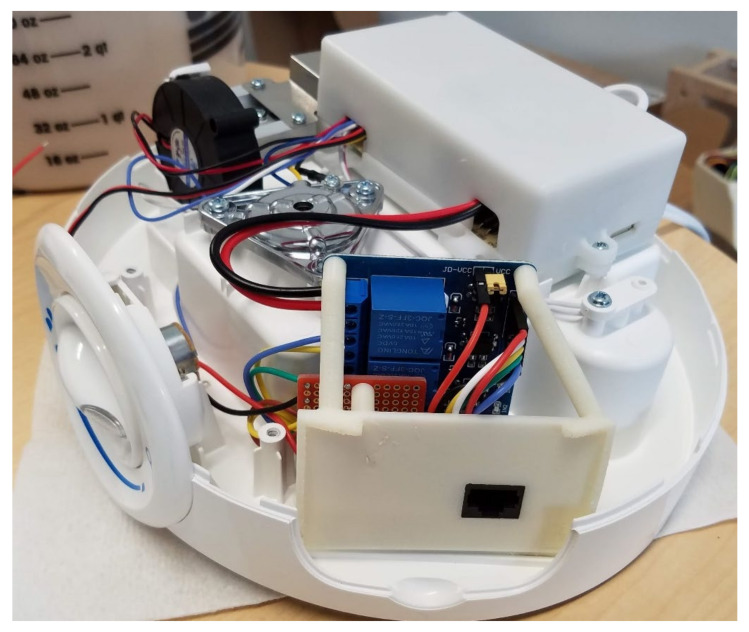
Assembled components used to automatically control the ultrasonic humidifier (bottom cover of humidifier removed for illustration).

**Figure 6:**
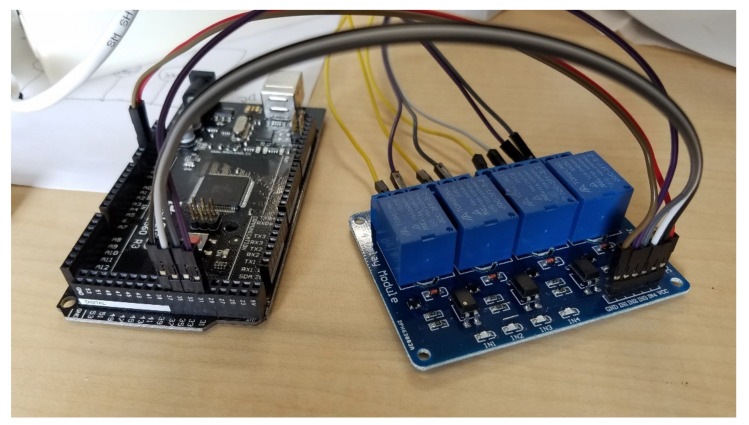
4-channel relay module connected to the Arduino MEGA 2560 microcontroller.

**Figure 7:**
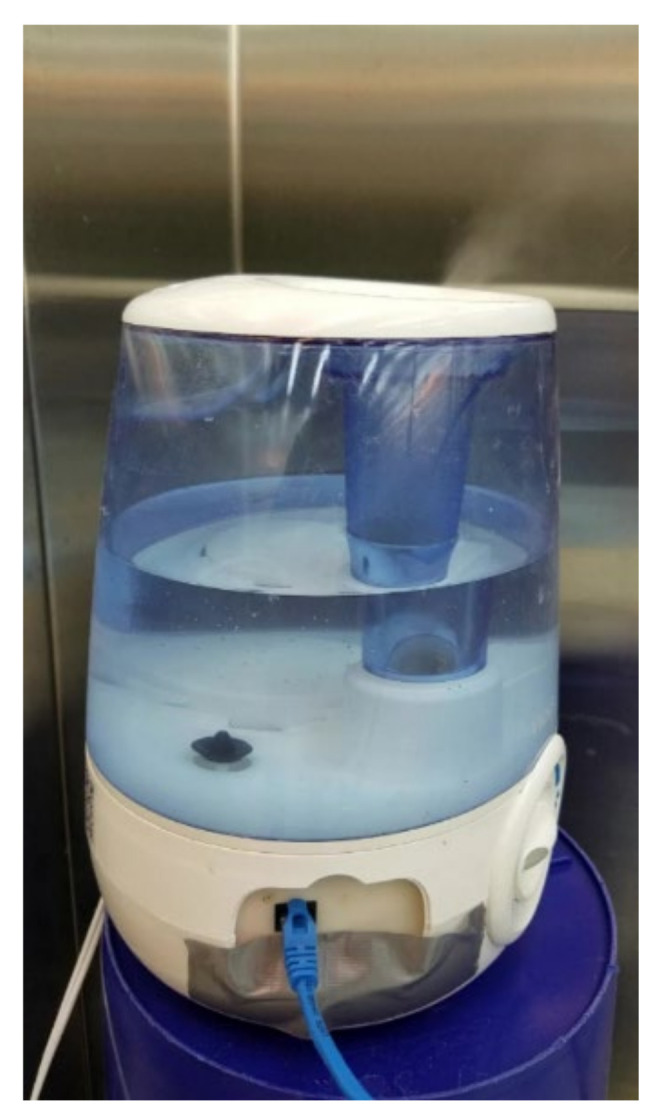
Assembled ammonia generator in operation.

The components were assembled and mounted to the ultrasonic humidifier as shown in Figure 5. The 4-channel relay module was connected to the Arduino MEGA 2560 microcontroller as illustrated in Figure 6 (relay pin IN1 to D31, IN2 to D33, IN3 to D35, and IN4 to D37). Figure 7 shows the assembled ammonia generator in operation. The individual parts used for the ammonia generator and control system are listed in Table 2.

### 2.6. Ammonia Generator Automatic Control

The Arduino microcontroller varied the output speed of the ammonia generator based on measurements from the ammonia sensor. Signal noise was smoothed in real-time using a cumulative moving average filter. The length of the period used for the moving average filter could be adjusted, with a longer period resulting in a smoother signal that reacted more slowly to changes in measured concentration. A shorter period allowed for quicker reaction to changes in ammonia concentration but resulted in a less steady signal. The size of the step change (ppm) was also adjustable and used to change the output speed of the ammonia generator. A smaller step size allowed for the finer control of generated ammonia concentration when using a weaker ammonia solution, and a larger step size allowed the control system to react to more rapid increases in ammonia concentration from using a stronger ammonia solution.

### 2.7. Preliminary Testing

The automatically controlled ammonia generator was connected to the microcontroller using an ethernet cable. One ammonia generator was configured to continuously operate at speed 5 to generate a steady background level of ammonia gas in the chamber by replacing the manual speed control potentiometer with a 340 Ω resistor. The automatically controlled ammonia generator was operated at the same time to generate the additional gas needed to reach the target concentration of 50 ppm.

Preliminary ammonia generator testing was performed using 2% ammonia liquid to determine the maximum concentration that could be produced from one or two ammonia generators operating continuously at maximum speed. The ammonia generators were placed inside the controlled environment chamber and were both filled with 3.8 L (1.0 gallon) of 2% ammonia liquid. The ammonia generators were then switched on and operated at full speed continuously. A highly accurate analyzer (model 7000 Fourier Transform Infrared (FTIR) analyzer, California Analytical Instruments, Orange, CA, USA) was used to document ammonia gas concentration inside the chamber. Measured data were recorded every minute onto a laptop PC.

### 2.8. Ammonia Sensor Evaluation

Three MQ-137 metallic oxide semiconductor (MOX) ammonia sensors were evaluated for accuracy relative to the highly accurate FTIR analyzer in the controlled environment chamber. The FTIR analyzer was located in a laboratory across a hallway from the controlled environment chamber. A gas sampling tube was located in the front left corner of the chamber, 0.3 m (1.0 ft) above the floor. An air sample was drawn continuously from the chamber at a rate of 5.0 l/min (0.18 cfm) through the FTIR using an air sampling pump (model 52669, Gast Manufacturing, Benton Harbor, MI, USA). The three MQ-137 sensors were mounted in a sealed 3D printed plastic housing located in the laboratory, with one end connected to the FTIR outlet port and the other end connected to tubing returning the sample air to the chamber to expose the sensors to the same air sample as the FTIR. The MQ-137 sensors were turned on and allowed to warm up for several hours until stable readings were attained in fresh air (no ammonia gas) before testing. The FTIR analyzer was turned on, and the sampling pump was operated to purge the analyzer using fresh air for 30 min before testing.

Two ammonia generators were placed in the controlled environment chamber along opposing side walls at the center of the room and spaced 0.3 m (1.0 ft) away from the walls. The ammonia generators were each filled with 3.8 L (1.0 gallon) of 2% ammonia cleaning liquid, then they were operated continuously at full speed to produce ammonia gas inside the chamber. A laptop PC logged the measured FTIR ammonia concentration every minute, and the MQ-137 sensor readings were recorded by a custom datalogger every five seconds. A handheld multi-gas meter (Model MX6, Industrial Scientific, Pittsburgh, PA, USA) was placed in the chamber for several minutes to compare measurements from its electrochemical ammonia gas sensor to the FTIR analyzer. Ammonia sensors were evaluated when the environmental chamber temperature was 26.7 °C (80 °F) and the relative humidity was 15%. Sensor measurement comparisons were made during a six-hour period when ammonia, temperature, and humidity levels were steady.

### 2.9. Ammonia Generator Testing Startup Procedure

The ammonia generator system was tested in the controlled environment chamber to evaluate its ability to maintain a desired setpoint ammonia concentration of 50 ppm and to determine how long the ammonia generators could operate before running empty. Prior to each test, the datalogger was placed in the chamber and powered on, and the MQ-137 ammonia sensor was operated in fresh air for several hours to allow the sensor to warm up and produce stable measurements. Next, the ammonia generator was operated to produce ammonia gas inside the chamber until the measured concentration reached 50 ppm measured with the handheld MX6 gas monitor as a reference. The MQ-137 sensor calibration was then adjusted to match the MX6 measurement.

### 2.10. Ammonia Generation with 2% Ammonia Liquid

The ammonia generator system was first evaluated using 2% ammonia cleaning liquid. Two ammonia generators were operated together in the controlled environment chamber. The first ammonia generator was configured to operate at speed 5 (of 8 speeds) continuously to generate a steady background level of ammonia gas in the chamber. The second ammonia generator was automatically controlled at the same time to generate the additional gas needed to reach the target concentration of 50 ppm. Each ammonia generator was filled with 3.8 L (1.0 gallon) of 2% ammonia cleaning liquid. The background ammonia generator was placed 0.3 m (1.0 ft) from the right side wall at the center of the chamber, the automatically controlled ammonia generator was placed 0.3 m (1.0 ft) from the left side wall at the center of the chamber, and the custom datalogger with the MQ-137 ammonia sensor was located in the front left corner of the chamber, 0.3 m (1.0 ft) above the floor. The ammonia concentration was recorded by the datalogger every five seconds until both ammonia generators ran empty. During the 2% ammonia liquid ammonia generator evaluation, the temperature and relative humidity in the controlled environment chamber were set to 26.7 °C (80 °F) and 50%, respectively.

A moving average filter period of 30 was used in the control system with 2% ammonia liquid to act as a signal damper for the rapidly fluctuating ammonia measurement signal. Ammonia generator output speed was controlled using an increment of 0.25 ppm. The generator was started at the highest speed until the measured ammonia concentration reached 48.75 ppm, then speed was decreased one step for every 0.25 ppm increase in concentration. The ammonia generator was stopped if the measured concentration reached 50.5 ppm.

### 2.11. Ammonia Generation with 10% Ammonia Liquid

The ammonia generator system was also evaluated using janitorial strength 10% ammonia cleaning liquid. Only one automatically controlled ammonia generator was used for evaluation with 10% ammonia liquid. The ammonia generator was filled with 3.8 L (1.0 gallon) of 10% ammonia liquid. The automatically controlled ammonia generator was placed 0.3 m (1.0 ft) from the right side wall at the center of the chamber, and the datalogger and MQ-137 ammonia sensor were located on the opposite side of the chamber, 0.3 m (1.0 ft) from the left side wall. The ammonia concentration was recorded by the datalogger every five seconds until the ammonia generator ran empty. During the 10% ammonia liquid ammonia generator evaluation, the temperature and relative humidity in the controlled environment chamber were set to 20 °C (68 °F) and 50%, respectively.

A smaller moving average filter period was used in the control system to allow the single generator to react more quickly to changes in ammonia concentration. The filter period was changed from 30 (used for 2% ammonia liquid) to 5. The control levels used to change speeds had to be adjusted from 0.25 to 2.0 ppm increments, starting at full speed until the concentration reached 36.5 ppm, then decreasing by one step for every 2.0 ppm increase and stopping at 50.5 ppm.

### 2.12. Continuous Operation during Six Week Study

After evaluation using 10% ammonia liquid, two ammonia generators were operated continuously for six weeks to maintain an ammonia gas concentration of 50 ppm in two controlled environmental chambers for a poultry health study. Each chamber was equipped with a single ammonia generator filled with 10% ammonia liquid and placed 0.3 m (1.0 ft) from the right side wall at the front of the chamber, with the datalogger and MQ-137 ammonia sensor located 0.3 m (1.0 ft) from the left side wall at the center of the chamber. The control system was configured in the same manner as during the 10% ammonia liquid evaluation, with a moving average filter period of 5 and 2.0 ppm control level increments used to change speeds, starting at full speed until the concentration reached 36.5 ppm, then decreasing by one step for every 2.0 ppm increase and stopping at 50.5 ppm. Although the ammonia generators were capable of operating for longer using only 3.8 L (1.0 gallon) of 10% ammonia liquid, the reservoirs were topped off daily as part of routine chamber cleaning and maintenance. Chamber temperature was set to 33.3 °C (92 °F) and gradually reduced to 20.0 °C (68 °F) over the six-week period. Relative humidity was not controlled. To minimize the effects of humidity on ammonia sensor measurements, the MX6 handheld gas monitor was used twice a day (morning and afternoon) to check the ammonia concentration inside the chambers, and MQ-137 calibration values were adjusted appropriately.

### 2.13. Safety

All personnel who entered the controlled environment chamber during ammonia testing used fitted half-latch respirators equipped with P100 cartridges approved for protection against ammonia gas. Only brief entry into the chamber during testing was allowed to place and retrieve a handheld MX6 personal gas monitor when performing occasional ammonia concentration measurement spot checks. The respirator was also worn when handling ammonia liquid since some gas is released when pouring it from the bottle into the ammonia generator reservoir.

## 3. Results

### 3.1. Preliminary Results

A single ultrasonic ammonia generator produced an ammonia gas concentration inside the chamber between 35 and 45 ppm when operated continuously on the highest speed setting using store bought 2% liquid cleaning ammonia. With two ultrasonic generators operating at the same time a steady concentration of 65 ppm was achieved.

### 3.2. Ammonia Sensor Evaluation

**Figure 8:**
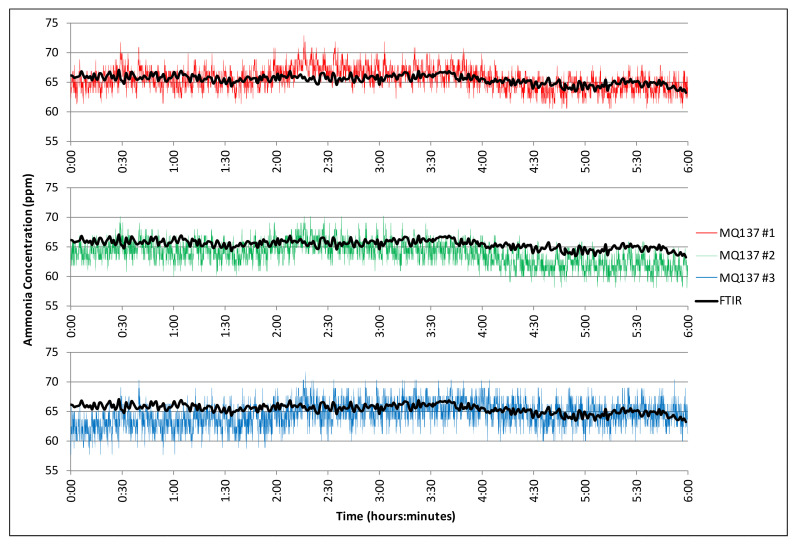
Measured ammonia gas concentration (ppm) over time from three metallic oxide MQ-137 sensors compared to the highly accurate FTIR.

The ammonia gas concentration recorded from three MQ-137 ammonia sensors (raw values) and the FTIR analyzer during a six-hour period with steady temperature (26.7 to 27.1 °C) and humidity (15.0% to 15.8%) is presented in Figure 8. The average concentration measured by the FTIR during this period was 65.4 ppm. The average MQ-137 sensor measurements over the six-hour period were within 1.4% of the FTIR. The measured ammonia gas concentration measured over a shorter period of several minutes using the MX6 multi-gas meter was within 7.9% of the FTIR (Table 3).

### 3.3. Evaluation Using 2% Ammonia Liquid

Using store-bought 2% ammonia cleaning liquid, one ammonia generator was operated continuously at speed 5 while the automatically controlled generator maintained a target level of 50 ppm inside the chamber. It took 30 min to raise the ammonia gas concentration inside the chamber from 0 to 50 ppm. The system was able to maintain the target concentration inside the chamber for a duration of 30 h before the generators ran empty.

**Figure 9:**
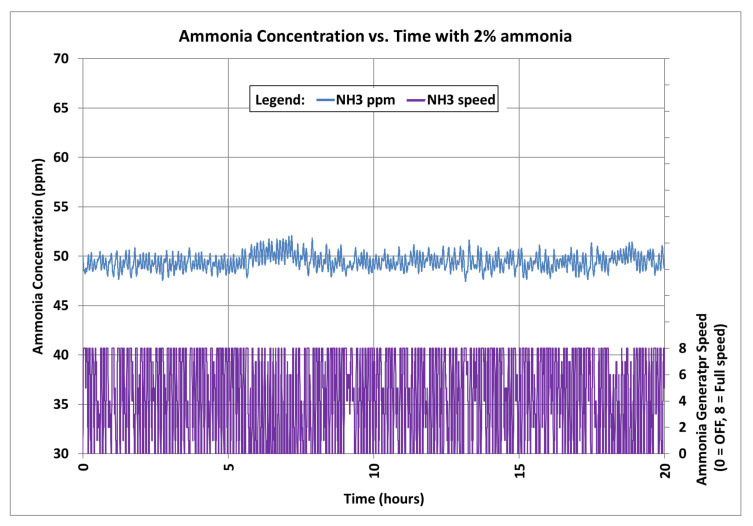
Chamber ammonia concentration (ppm) and automatically controlled ammonia generator speed over a 20-h period (with background generator speed = 5) using 2% ammonia liquid.

The average temperature and relative humidity were 26.7 °C (80.1 °F) and 47.8%, respectively, over the duration of the test. Since the upper humidity limit inside the controlled environment chamber was not controlled, the statistical analysis for the ammonia control system was performed over a 20-h period when the temperature and humidity in the controlled environment chamber were steady, from 26.5 to 26.9 °C (79.7 to 80.4 °F) and 46.7% to 50.8%, respectively. The ammonia gas concentration maintained inside the chamber during this 20-h period was 49.45 ± 0.79 ppm (*n* = 15,246) (Figure 9). During this time, the automatically controlled ammonia generator was stopped 26% of the time, operated at full speed 31% of the time, and operated at each of the other speeds fairly evenly from 5% to 7% of the time.

### 3.4. Evaluation Using 10% Ammonia Liquid

In preliminary tests using 10% ammonia liquid, both the automatically controlled and the background generators were used, and 1.9 L (0.5 gallon) of liquid was added to each generator. The speed of the background generator was still set to 5, as during the 2% ammonia liquid evaluations, and the automatic generator was programmed to turn off when the ammonia gas concentration reached 50.5 ppm. However, the background generator using 10% *v/v* ammonia solution was able to produce an ammonia concentration inside the chamber of 300 ppm after only five minutes of continuous operation at speed 5. It was thus determined that with the 10% *v/v* ammonia solution, the background generator would not be needed.

After adjustments to the control algorithm for the stronger ammonia solution, the test was performed using only the automatically controlled ammonia generator. The reservoir was filled with 3.8 L (1.0 gallon) of 10% ammonia liquid with the generator set to maintain an ammonia gas concentration of 50 ppm in the controlled environment chamber. It took 17 min to raise the ammonia gas concentration inside the chamber from 0 to 50 ppm. The single automatically controlled ammonia generator maintained an ammonia concentration of 50 ppm for 195 h when using 10% ammonia liquid. The average temperature and relative humidity were 19.7 °C (67.5 °F) and 44.1%, respectively, over the duration of the test.

**Figure 10:**
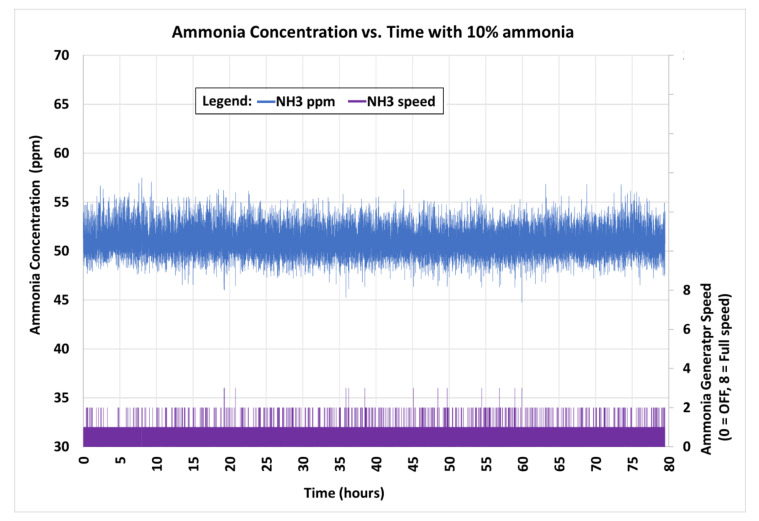
Chamber ammonia concentration (ppm) and generator output speed over an 80-h period from one generator using 10% ammonia liquid.

Since the upper humidity limit inside the controlled environment chamber was not controlled, statistical analysis for the ammonia control system was performed over an 80-h period when the temperature and humidity in the controlled environment chamber were steady, from 19.4 to 19.9 °C (66.9 to 67.8 °F) and 42.3% to 54.3%, respectively. The ammonia gas concentration maintained inside the chamber during this 80-h period was 51.24 ± 1.53 ppm (*n* = 47,477) (Figure 10). During this time, the ammonia generator was stopped 66% of the time, operated at speed 1 during 32% of the time, and operated at speed 2 only 2% of the time. The ammonia generator did not operate at higher output speeds using 10% ammonia liquid.

### 3.5. Continuous Operation during Six-Week Study

The ammonia generators using 10% ammonia liquid were operated continuously for six weeks to maintain a concentration of 50 parts per million (ppm) of ammonia gas in two controlled environment chambers for a poultry health study. The temperature inside the chambers started at 33.3 °C (92 °F) and was gradually reduced to 20.0 °C (68 °F) over the six-week period. The relative humidity level inside the chambers varied between 11 and 37% due to outdoor conditions (mean RH = 21.1 ± 6.5%), so frequent calibration adjustments of the ammonia gas sensors was necessary (twice a day).

During the six-week experiment, the ammonia generators maintained an average ammonia concentration in the two controlled environment chambers of 46.42 ± 3.81 ppm (*n* = 597,815 measurements) and 45.63 ± 4.95 ppm (*n* = 556,609 measurements).

## 4. Discussion

### 4.1. Selection of the Ammonia Sensors

The MQ-137 sensor was selected because it could be used to detect ammonia gas continuously in the range of 5–500 ppm and was not sensitive to other common gases that would be present in the controlled environment chamber. The other gases the MQ-137 sensor is sensitive to are carbon monoxide (CO) and dimethyl ether (C_2_H_6_O), neither of which should be present in the controlled environment chamber during animal health experiments.

### 4.2. Ammonia Generation

Based on the evaluation of the ammonia generator using 10% ammonia liquid in a controlled environment chamber, we expected continuous operation of one ammonia generator for up to 195 h using 3.8 L (1 gallon) of 10% ammonia liquid before refilling was required, which equates to an average ammonia liquid consumption rate of 0.45 L (0.12 gallons) per day. However, at the end of the six-week experiment, two ammonia generators operating continuously for six weeks to maintain an ammonia concentration of 50 ppm in two separate controlled environment chambers consumed a total of 200 L (53 gallons), or approximately 100 L (26.5 gallons) for each chamber at an average consumption rate of 2.4 L (0.63 gallon) per day. Several factors may have contributed to this large difference in actual versus expected ammonia liquid usage. The actual concentration (% *v*/*v*) of ammonia within each bottle may be affected by age and storage conditions. Although we did not measure the ammonia concentration (% *v*/*v*) of each bottle, we anticipate that variation in the strength of ammonia liquid can result in variation in operating time before refilling the ammonia generator reservoir is necessary. In addition, during the 195-h evaluation, the controlled environment chamber was undisturbed, and the average indoor relative humidity level was 44%. However, during the six-week animal health study, animals were present in the chambers, and the chamber doors were routinely opened several times a day for animal husbandry and chamber maintenance and to refill the ammonia generators. Additionally, due to technical problems with the chamber humidifiers, the humidity inside the chambers averaged only 21% during several days. Further study is required to determine if large differences in environmental humidity influences the rate of consumption of ammonia liquid.

Regardless of variations in the concentration of ammonia liquid, the ultrasonic ammonia generator will automatically operate at higher or lower speeds to reach the setpoint concentration by design. This feature may be even more convenient when used in a chamber with birds raised on litter wherein the manure is releasing some of the ammonia gas. Our automatic system should be able to compensate for the ammonia emitted from the litter as long as the total of the released ammonia plus the ammonia that is generated by the background ammonia generator does not exceed the target setpoint. If the ammonia released by the litter is substantial, the removal of the background ammonia generator should be considered.

Throughout the experiments, we noticed that the MQ-137 ammonia readings are very susceptible to changes in environmental humidity. For this reason, we had to recalibrate our systems twice each day. Due to the influence of humidity on ammonia sensor readings, future refinements to the custom datalogger will include real-time adjustments to ammonia sensor measurements based on measured changes in air temperature and relative humidity.

### 4.3. Suitability of the MQ-137 Sensors for Medium to Long-Term Measurement of Ammonia Concentration

The three MQ-137 sensors we tested demonstrated just a small deviation from the FTIR analyzer. This indicates that, despite their low cost, they seem to be consistent instruments. In addition, after well over 1000 h of operation in contact with ammonia gas, they continued to operate without much deviation from the readings provided by the MX6 handheld meter. This makes the MQ-137 sensors suitable for long-term applications in presence of ammonia gas at levels of 50 ppm.

### 4.4. Variability of the Tested Systems

During controlled testing, the systems maintained an ammonia gas concentration of 49.45 ± 0.79 ppm using two ammonia generators filled with 2% ammonia liquid [33] and 51.24 ± 1.53 ppm using one generator filled with 10% ammonia liquid [34]. When using the 10% ammonia liquid, a careful calibration of the control algorithm (ppm step change and moving average filter period) was needed to maintain the concentration of ammonia within a tight range. Even with those changes in place, the system using 2% ammonia liquid maintained the target ammonia gas concentration of 50 ppm with less variation compared to the 10% ammonia liquid. The main reason for the high variation using 10% ammonia liquid was the high concentration of gas produced even at the lowest speeds of the generator. Using a system with 10% ammonia solution may be preferable when concentrations higher than 50 ppm are needed in a chamber of similar size. However, when more accuracy is desired, a weaker solution of liquid ammonia will maintain the target levels within a narrower range. Tighter control using 10% ammonia in one generator may also be possible by changing resistor values in the relay control board so that the full range of generator output speed can be utilized.

During six weeks of testing with animals, the system maintained 46.42 ± 3.81 ppm and 45.63 ± 4.95 ppm in two controlled environment chambers when operated continuously using 10% ammonia liquid. This is a lower average concentration than achieved during the 195-h evaluation, but the relative humidity inside the two chambers was not controlled. Many other factors may have caused variability for the system during the six-week study, such as daily maintenance inside the chambers; refilling drinking water troughs (increasing chamber humidity for a period of time); refilling the ammonia generator reservoirs, which releases high concentrations of ammonia gas for a short period of time; and wear of the humidifiers.

### 4.5. Continuous Operation for Six Weeks and Wear-and-Tear

The control system was initially configured with a moving average filter period of 5 and 2.0 ppm control level increments used to change speeds, starting at full speed until the concentration reached 36.5 ppm, then decreasing by one step for every 2.0 ppm increase and stopping at 50.5 ppm. After continuous operation for 21 days, the ammonia generators seemed to have some trouble reaching the target setpoint at the lowest speed of 1, so the control algorithm was changed and the lowest speed was not used for the remainder of the experiment. The control algorithm was modified to run at full speed = 8 until the concentration reached 38.5 ppm, decreasing by one step for every 2.0 ppm until speed = 2, then stopping when 50.5 ppm was reached. The subtle decline in ammonia generator output observed during the first three weeks of the animal health study could have been due in part to a weaker ammonia solution but may also have been due to wear and tear of the ultrasonic ammonia generators. After 30 days, the ammonia generator with the most runtime failed, and it was discovered that its ceramic ultrasonic disc was pitted. That generator was the first one developed and tested, and it had been used extensively for hundreds of hours previously when evaluating ammonia sensors and control system performance with 2% and 10% ammonia liquid. Therefore, it is reasonable to assume the wear and tear of the ceramic disc played a large role in the decreased ammonia generator output over time. When planning the six-week experiment, extra replacement components were purchased for just such an event, and exchanging the ammonia generators was a simple task that involved moving the custom control circuitry from the old units into the replacements. If suitable replacement ceramic ultrasonic discs are available, it may be possible to replace only the pitted disc to return the generator to service.

### 4.6. One vs. Two Generators

Using 2% ammonia liquid with two generators, it took 30 min to raise the ammonia gas concentration from 0 to 50 ppm inside the chamber, which had a volume of 38 m^3^ (1344 ft^3^) and an exhaust air flow rate of 0.06 m^3^/s (130 cfm). Using 10% ammonia liquid with one generator, it took 17 min to go from 0 to 50 ppm. How quickly the ammonia generation system reaches the setpoint concentration will depend on many factors, most importantly the size of the room and exhaust air flow rate. Variations in the strength of the ammonia solution will also have an effect, with weaker solutions requiring more time to reach a setpoint concentration of 50 ppm. Additional generators can be used for larger rooms or higher exhaust air flow rates. There was not a noticeable decrease in ammonia concentration inside the chambers when the doors were opened for brief durations of time during daily chamber maintenance because the ammonia generation system started to operate as soon as the concentration of ammonia fell below 50 ppm.

An advantage of using two generators with 2% ammonia liquid for animal health studies is redundancy. The ammonia concentration should not drop to zero if one generator fails since the second would continue to operate. If the automated generator fails, the background generator will still produce ammonia output at a constant speed level (speed = 5). If the background generator fails, the automated generator will operate up to full speed, if necessary, to try to maintain the setpoint concentration. A disadvantage of the two-generator setup with weaker ammonia solution is the need for more frequent refills. Extra capacity in the system using 10% ammonia means the generator can be operated at lower speeds for longer durations of time.

### 4.7. Ammonia Supply System Safety

Previous studies used compressed anhydrous ammonia gas cylinders with an electromechanical cutout valve to stop the supply of ammonia in case of a power failure. But this would not prevent dangerous levels of ammonia gas from building up inside the test chamber in the case of an incomplete power failure, which might occur if a ventilation system circuit breaker overloads or if a ventilation fan motor fails. The cutout valve would also not prevent leakage from loose fittings or cracked hoses. Our automatically controlled ammonia generator is safer for three reasons: 1. The ultrasonic disc would stop atomizing ammonia liquid if there was a power failure. 2. Since the ammonia generator only operates to supply ammonia until the desired setpoint concentration is reached, the ammonia supply would automatically stop in the case of a ventilation shutdown when the setpoint concentration gets reached, preventing excessive gas buildup. 3. The ammonia liquid used, 2 or 10% *v/v*, would not release dangerously high levels of ammonia if spilled. But a compressed gas cylinder filled with 99% anhydrous ammonia could easily fill a closed test chamber with toxic or even explosive concentrations of ammonia gas.

### 4.8. Cost Comparison to Gas Cylinder Systems

A comparison can be made between the cost for the automatically controlled ammonia generation system presented in this paper and the estimated cost to use compressed anhydrous ammonia gas for the same purpose. The cost estimate for the anhydrous ammonia delivery system is based on prices quoted prior to beginning the six-week research study and only includes the gas, cylinder, and flow regulator. The most expensive component when using compressed anhydrous ammonia gas is the flow regulator, which must be made of stainless steel because ammonia corrodes copper–zinc alloys such as brass. The cost for a stainless steel dual-stage flow regulator suitable for use with anhydrous ammonia gas was around $1200.00. The gas cylinder cost was $258.00 for a size K cylinder. Ignoring the cost of hoses or other fittings that may be needed, that makes the total cost of the anhydrous ammonia gas delivery system $1458.00. Based on the chamber volume, exhaust air flow rate, and assuming perfect mixing, it was estimated that 13.1 L of compressed anhydrous ammonia would be needed to maintain a concentration of 50.0 ppm inside one controlled environment chamber for a duration of six weeks at a cost of $21.25/liter, or $278.34.

The total cost for components to build the automatic ammonia generator was approximately $150.00. Estimated labor to assemble the components and modify one ultrasonic humidifier is around 8 h at a cost of $25.00/h, or $200.00. That makes the total cost for the ammonia generator system $350.00. The total cost for 100 L (26.5 gallons) of 10% ammonia liquid used for one chamber during the six-week study was $158.74.

Based on these estimates, the developed ammonia generation system can be built for one-quarter the cost of the gas cylinder supply system. The ammonia liquid cost for six weeks of continuous operation is also less expensive than using compressed anhydrous ammonia gas.

## 5. Conclusions

1. An inexpensive ammonia generator system was developed to maintain a constant setpoint ammonia gas concentration for animal health studies. The main components for building and running these systems, ultrasonic humidifiers and ammonia liquid, are inexpensive and readily available from many local stores.
2. The developed ammonia generation system could maintain a stable ammonia gas concentration of 50 ± 5 ppm inside the controlled environment chamber. Longer operational times between refreshing ammonia liquid can be achieved when using the 10% vs. the 2% ammonia solutions.
3. Inexpensive MQ-137 ammonia sensors presented high agreement in ammonia concentration when compared against a high-accuracy reference instrument. With frequent recalibration to account for changes in relative humidity, the MQ-137 sensors continued to operate reliably for over 1000 h of continuous use in the presence of 50 ppm of ammonia gas.
4. The system presents minimal safety concerns for animals and humans in comparison to systems using compressed ammonia gas.

## Figures and Tables

**Table 1:** Humidifier speed control circuit resistor values and relay Open/Closed states.

		R1	R2	R3
Speed	Total	470	220	100
	Ω	Relay 1	Relay 2	Relay 3
Slow 1	810	Open	Open	Open
2	710	Open	Open	Closed
3	590	Open	Closed	Open
4	490	Open	Closed	Closed
5	340	Closed	Open	Open
6	240	Closed	Open	Closed
7	120	Closed	Closed	Open
Full 8	20	Closed	Closed	Closed

**Table 2:** Parts list for automatically controlled ammonia generator and controller.

Quantity	Component
1	Vicks V4600 ultrasonic humidifier
1	RJ45 jack
1	RJ45 breakout board
1	Mini solder-able breadboard
1 each	Speed control resistors: 20, 100, 220, and 470 Ω
1	4-channel 5 V relay module
1	5VDC 700 mA power supply module
1	Arduino MEGA 2560 R3 microcontroller
1	RJ45 jack
1	RJ45 breakout board
1	DS3231 real-time clock module
1	MicroSD card module
1	16 GB MicroSD card
1	MQ-137 ammonia sensor
1	Ammonia sensor load resistor: 47 kΩ
1	9VDC 1500 mA power adapter
2	Female blade terminal crimp connectors
1	2-pin JST-XH male plug
2	Female JST-XH crimp pins
1	6-pin 2.54 mm pitch female connector housing
1	1-pin 2.54 mm pitch female connector housing
7	Female 2.54 mm pitch crimp pins
1	3D printed mount
6	M2 × 4 mm thread forming screws
Multiple	22-gauge silicone stranded wires
1	Ethernet cable

**Table 3:** Ammonia gas sensor concentration (ppm) comparison statistics during a six-hour period (*n* = 345 readings for FTIR, *n* = approximately 4320 readings per MQ-137 sensor, *n* = 10 readings for MX6).

Instrument	Average Ammonia Reading	Min	Max	Standard Deviation	Percent Error * vs. FTIR
FTIR	65.4	63.2	67.1	0.8	(reference)
MQ-137 #1	65.6	60.6	72.9	1.9	0.2%
MQ-137 #2	63.9	58.1	70.1	2.0	2.4%
MQ-137 #3	64.5	57.8	71.8	2.2	1.5%
MX6	71.0	71.0	71.0	0.0	7.9%

* Percent Error = $\frac{\;\left|\mathrm{Average}\;\mathrm{Sensor}\;\mathrm{ppm}\;-\mathrm{Average}\;\mathrm{FTIR}\;\mathrm{ppm}\right|}{\left|\mathrm{Average}\;\mathrm{FTIR}\;\mathrm{ppm}\right|}\times 100\%$.
